# Maltodextrin-Nanoparticles as a Delivery System for Nasal Vaccines: A Review Article

**DOI:** 10.3390/pharmaceutics16020247

**Published:** 2024-02-07

**Authors:** François Fasquelle, Angelo Scuotto, Michael Howsam, Didier Betbeder

**Affiliations:** 1 Vaxinano SAS, F-59120 Loos, Francedidier.betbeder@vaxinano.com (D.B.); 2 Université de Lille, Inserm, Centre Hospitalier de Lille, Institut Pasteur de Lille, U1167—RID-AGE—Facteurs de Risque et Déterminants Moléculaires des Maladies Liées au Vieillissement, F-59000 Lille, France

**Keywords:** maltodextrin, nanoparticles, nasal vaccine, antiparasitic vaccines, antiviral vaccines, antibacterial vaccines

## Abstract

Nanoparticles are increasingly being studied as antigen delivery systems for immunization with nasal vaccines. The addition of adjuvants is still generally required in many nanoparticle formulations, which can induce potential side effects owing to mucosal reactogenicity. In contrast, maltodextrin nanoparticles do not require additional immunomodulators, and have been shown to be efficient vaccine delivery systems. In this review, the development of maltodextrin nanoparticles is presented, specifically their physico-chemical properties, their ability to load antigens and deliver them into airway mucosal cells, and the extent to which they trigger protective immune responses against bacterial, viral, and parasitic infections. We demonstrate that the addition of lipids to maltodextrin nanoparticles increases their potency as a vaccine delivery system for nasal administration.

## 1. Introduction

The development of nasal vaccines began in the early 1990s with work on sub-unit antigens by Berna Biotech, Switzerland, and Biovector Therapeutics, France, as the pioneers. The use of live attenuated vaccines presents a risk of reversion and compromises their safety after mucosal administration, as observed for the oral polio vaccine [1]. Hence, inactivated vaccines (subunit antigens, recombinant proteins, etc.) have become the main alternative, and these are often associated with adjuvants to enhance their immunogenicity [2,3]. Nonetheless, early studies showed that the nasal mucosa has a high reactogenicity against many adjuvants, owing to the triggering of local inflammation, and that some adjuvants might migrate also to the brain [4,5]. For example, Berna Biotech used a modified *Escherichia coli* enterotoxin immunomodulator in their Nasalflu, and subsequent studies revealed that the adjuvant significantly increased the risk of developing Bell’s palsy, an illness that causes temporary facial paralysis [6,7].

The use of a less reactogenic delivery system utilizing nanoparticles (NPs) has since been widely investigated as an alternative to these adjuvants. Their ability to be loaded with the antigens, to facilitate antigens’ crossing of mucosal barriers, and then to be delivered directly to the nasal associated lymphoid tissue (NALT) increases the immunogenicity of subunit vaccines [8,9]. Consequently, NP delivery systems have emerged as an important and promising field in vaccine research.

The NP systems designed for nasal vaccine delivery have been developed from a wide range of molecules and polymers. Among them, natural polysaccharides and phospholipids are the most commonly used thanks to their biocompatibility (ensuring a low reactogenicity) and the ease with which they can be chemically modified [10,11]. Maltodextrins are natural polysaccharides extracted from starch and can be chemically modified to design NPs for various purposes, though they have rarely been exploited for vaccine delivery [12,13]. Nevertheless, over the last 3 decades, they have been shown to be efficient delivery systems for nasal vaccines against various diseases, thanks partly to their physicochemical properties.

In this article, we will focus upon this class of NPs, describing recent progress made in the development of maltodextrin NP-based systems for nasal vaccines for both human and animal applications.

## 2. The Use of NPs in Nasal Vaccines

The principle of a mucosal delivery system is to compensate for the low immunogenicity of subunit vaccines, but a vector is needed to facilitate their passage across mucosal barriers and then deliver the antigens directly into mucosal immune cells [14]. Emerging from technologies derived from particle physics and chemistry, nanosized vectors are capable of both encapsulating antigens and interacting with the immune cells. Polysaccharide-based NPs, polylactic-co-glycolic acid (PLGA) NPs, liposomes and acrylamide NPs have been the most widely used [8,15], though the adjuvants are generally associated with the antigens to achieve the required efficacy [16,17]. Some NPs used in vaccines are intrinsically immunogenic, depending on the starting material used for their synthesis, but may trigger too much inflammation to be administered across mucus membranes [18]. In this review, we focus on maltodextrin NPs as an efficient antigen delivery system without the use of immunomodulators for vaccines.

**Figure 1:**
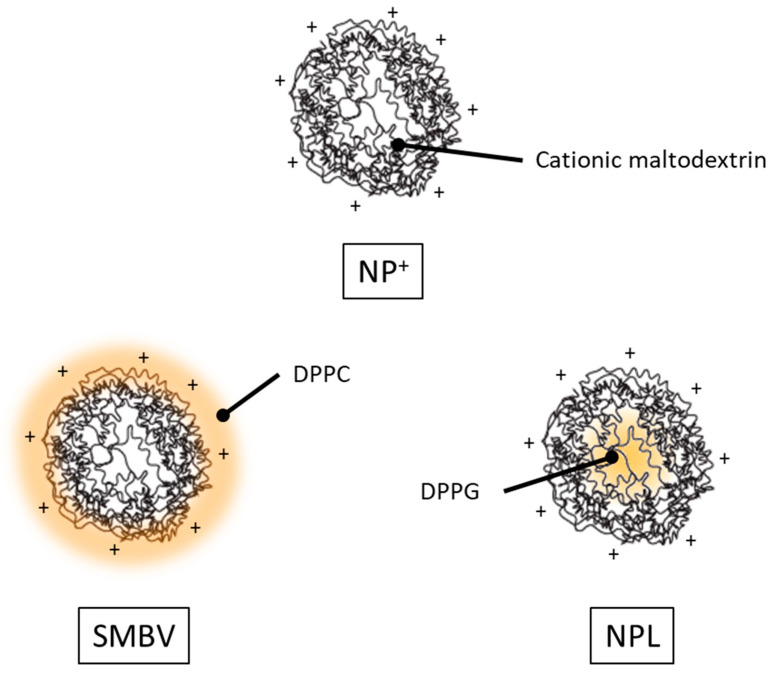
Schematic representation of the different maltodextrin NPs developed for nasal vaccination. NPs^+^ are NPs made from reticulated and cationized maltodextrin; SMBVs are NPs^+^ covered with a zwitterionic phospholipid bilayer; NPLs are NPs^+^ with an anionic phospholipid core.

The study of maltodextrin nanoparticles started in the early 1990s at Biovector Therapeutics under the direction of Didier Betbeder [19]. The idea was to synthesize NPs that mimic the way in which viruses cross the mucous barrier and are capable of delivering antigens into mucosal immune cells. Notably, their synthesis was performed in aqueous solvent according to the principles of green chemistry. Moreover, maltodextrin is an FDA-approved polysaccharide [20]. This simplified their manufacture, avoided ecological issues, contrary to maltodextrin NPs produced via oil-in-water emulsification [21,22,23] or nanoprecipitation [24]. Importantly, they also avoided the toxicity induced by vestigial organic solvents or surfactants, as has often been observed with NP synthesis [25]. Different kinds of maltodextrin NPs were synthetized based on this approach, and then used in the development of nasal vaccines: the three main types are shown in Figure 1.

The original NPs are produced from starch maltodextrin, the polysaccharide being solubilized in NaOH and cationized with quaternary ammonium (GTMA) before reticulation with epichlorohydrin. The epichlorohydrin that does not covalently bind to the maltodextrin chains is neutralized by the hydroxide ions, leading to the formation of glycerol. The cationic gel thus obtained is then crushed until all particles are less than 100 nm in size, before being purified via ultrafiltration to eliminate all salts and synthesis residues. The resulting NPs^+^ are porous with a cationic surface charge, enabling them to be taken up by antigen-presenting cells (APCs) but also by non-phagocytic cells. These NPs^+^ can be further covered by a phospholipid bilayer to obtain a version called supramolecular biovectors (SMBVs, Figure 1), or else loaded with anionic phospholipids to produce lipidated nanoparticles (NPLs, Figure 1).

## 3. NPs^+^ as a Vaccine Delivery System

### 3.1. Protein Loading

NPs^+^ have cationic charges distributed not only on their surface but in the entire maltodextrin scaffold. Nevertheless, the characterization of the interfacial structure showed an external, highly cationic layer and an internal layer with a lower density of charge [26]. Hence, NPs^+^ can be loaded with proteins by means of “post-loading”, i.e., mixing premade nanoparticles in water with proteins in a solution, exploiting electrostatic interactions between the anionic charges of the proteins and the cationic charges of the NPs^+^.

The extent of loading can be evaluated precisely using non-denaturing polyacrylamide electrophoresis gel (native PAGE). In contrast with free protein, NPs are too large to migrate within the gel and the protein bound by the NPs^+^ therefore cannot migrate; by comparing the migration of protein with or without NPs, it is possible to measure the percentage of loaded protein.

Using bovine serum albumin (BSA), NPs^+^ were shown to be loadable with 200% of their weight of proteins, displaying an encapsulation efficiency of 100% [26]. The absence of a significant decrease in zeta-potential or increase in size when mixing NPs^+^ with the proteins indicates an encapsulation rather than a binding of proteins on the particle surface [26]. By comparison, the encapsulation efficiency of BSA in liposome, PLGA or chitosan nanoparticles has been reported to be 10%, 60% and 70%, respectively [27,28,29].

### 3.2. Cell Interaction

The surface charge plays an important role in NPs, interaction with cells. Thus, compared with neutral or negatively charged NPs, cationic NPs exhibit a greater uptake by cells due to electrostatic interactions with the negative charges on the cell surface [30,31,32]. The ability of NPs^+^ to be endocytosed has been evaluated and confirmed in airway epithelial cells.

The NP^+^ surface charge decreases slightly in the presence of serum, but their size does not increase. This suggests the formation of a protein corona, which partly masks the cationic charge of the NPs^+^ but does not provoke any aggregation. Other NPs (inorganic NPs, PLGA, chitosan, etc.) have been shown to aggregate when incubated in culture media [33,34]. Aggregation results in the uncontrolled formation of a heterogenic population of nano- and microparticles with physicochemical properties different from those of exclusively nanoscale NPs [35].

**Figure 2:**
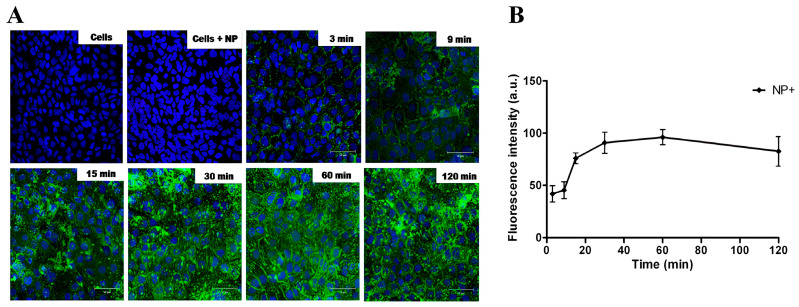
Uptake kinetics of NPs^+^ in 16HBE cells visualized by confocal microscopy. Cells were incubated at 37 °C with 10 μg of FITC-labeled NPs^+^ (green), for 3, 9, 15, 30, 60 or 120 min, fixed with 4% PFA, and nuclei were stained with TOPRO-3 (blue). (**A**) Endocytosis in each condition was visualized by confocal microscopy; scale bar = 50 μm. (**B**) Amount of endocytosis was then quantified from pictures fby measuring fluorescence intensities [36]. Reprinted with permission from [36]. Copyright 2023 IOP Publishing: Bristol, UK.

Importantly, the protein corona associated with NPs^+^ does not hinder their uptake by cells. Fluorescent microscopy studies performed in bronchial epithelial cells (16HBE14o-) revealed that most cells were fluorescent after 3 min of incubation, and NPs were found within all cells after 15 min (Figure 2, [36]). Mechanistic studies using endocytosis inhibitors revealed that NPs^+^ are endocytosed by clathrin pathways only. However, colocalization analysis by means of confocal microscopy revealed that they remain in the endocytosis vesicles, without any trafficking to intracellular organelles. Surprisingly, exocytosis was also observed, governed by a cholesterol-mediated pathway.

Regarding their genotoxicity toward epithelial cells, NPs^+^ do not induce a significant increase in DNA damage, even at high doses, contrary to other cationic NPs, probably due to the fact that they do not accumulate within cells as a result of their exocytosis [26,37].

### 3.3. Antigen Delivery

Hence, NPs^+^ can encapsulate proteins in their core without completely masking their cationic surface charges, meaning that they can still interact with the cell membrane via electrostatic interactions. No difference of endocytosis was observed in epithelial cells between empty NPs^+^ and NPs^+^ loaded with ovalbumin (OVA) [36]. The NP^+^ uptake and protein delivery were observed after just 3 min of incubation, with endocytosis saturation after 30 min. The protein delivery and processing by the cells was confirmed with the delivery of dye-quenched ovalbumin (OVA-DQ), with an increasing fluorescence observed after 30 min incubation.

To sum up, NPs^+^ loaded with proteins are first endocytosed by epithelial cells via a clathrin pathway; then, the NPs^+^ release the antigens in the cells, and they are finally exocytosed via a caveolae-dependent pathway.

### 3.4. NPs^+^ as a Delivery System for Mucosal Immunization

The ability of NPs^+^ to induce tolerance to allergens after sublingual administration has been studied. NPs^+^ were first loaded with OVA, representing an allergen, and the formulation was administered via the sublingual route [38]. Immunohistology analysis showed that after 5 min, OVA was found on the surface of mucosal tissues, and that it then penetrated deeper into the cells after 30 and 60 min. The protein was indeed captured and processed by local APCs, mainly dendritic cells (DCs). These DCs then migrated to afferent lymph nodes, promoting an immune tolerance toward the antigen by stimulating both IFN-γ- and IL-10-secreting CD4^+^ lymphocytes. A second study was performed on birch pollen allergic asthma, with NP+ being loaded with a recombinant form of the major allergen Betv1a, again administered via the sublingual route. A significant reduction in chronic airway hyperresponsiveness, lung eosinophilia and Th2 responses were induced compared to administering the free antigen. These results highlight the efficiency of NP+ as a mucosal delivery system, which triggers a Th2 immune response more favorably than a Th1 response after nasal immunizations. This work formed the platform upon which further research with variants of NP+ were developed.

## 4. Lipidated Cationic Maltodextrin NPs for Antigen Delivery

Lipidated cationic maltodextrin NPs were designed, based upon NPs^+^, in order to create a delivery system able to load a wider range of antigens, including hydrophobic molecules, and to deliver them more efficiently into the cytosol. Hence, NPs^+^ were either coated with amphiphilic phospholipids to produce SMBVs or filled with anionic phospholipids to produce NPLs (Figure 1). The main differences between each particle are summarized in Table 1.

### 4.1. SMBVs

SMBVs were developed by Biovector Therapeutics in the early 1990s, the aim being to mimic the physicochemical characteristics of enveloped viruses such as influenza or human immunodeficiency virus-1 (HIV) [39,40]. The phospholipid membrane coating of these nanoparticles consisted of dipalmitoyl phosphatidyl choline (DPPC) and cholesterol, and it was hypothesized that this would improve their loading capacity for membrane proteins as well as their adherence to mucosal surfaces. The resulting NPs exhibited a neutral surface charge, due to the covering of the NP by the lipid membrane. The phospholipids were organized in a single bilayer, and no significant size increase was observed compared with NPs^+^. Contrary to DPPC liposomes, SMBVs remained stable in a solution for 6 months at room temperature, and for at least a year at 4 °C [41].

The formation and stability of SMBVs can be explained by electrostatic interactions between the phosphate groups of DPPC and the cationic charges of the NPs^+^. Strong carbohydrate–phospholipid interactions are also likely to happen through the establishment of hydrogen bond networks between water, sugars and phospholipids [42,43,44].

#### 4.1.1. Antigen Loading

Their dual structure permits SMBVs to be loaded with molecules with different physicochemical features: the cationic polysaccharidic core can encapsulate anionic molecules, as observed for NPs^+^, while the external phospholipid layer can adsorb membrane proteins.

A chimeric protein GST-e4, resulting from the fusion of Escherichia coli glutathione-S-transferase (GST) with the 406 amino acid C-terminal fragment (e4) of protein IE1 from the human cytomegalovirus (hCMV), was associated with NPs^+^ and SMBVs. In both cases, 90% encapsulation of GST-e4 was attained, confirming that the lipid covering does not hinder the post-loading procedure. The formulation was found to be stable over time and the protein was protected from enzymatic degradation [41].

In other studies, the association of the human recombinant interleukin-2 (hrIL-2) with SMBVs increased its proliferative activity toward an IL-2-dependent cytotoxic T-lymphocyte line. Interestingly, an impaired hrIL-2 associated with SMBVs was able to recover its biological activity, probably due to the association with the lipid membrane of SMBVs [41].

Further studies have been carried out on oligodeoxynucleotides (ODNs) and showed that SMBVs could be loaded with at least 10% ODN (*w*/*w*) with 100% of encapsulation efficiency [45]. This encapsulation was again highly stable, as no size increase of the formulation occurred over time, and no ODN leakage was observed in a growth medium supplemented with 10% FBS.

#### 4.1.2. Cell Interaction and Antigen Delivery

Despite their apparently neutral surface charge, SMBVs are likely to interact with the cell membrane thanks to their cationic charge, and therefore be endocytosed to deliver antigen within cells.

The in vitro studies with SMBVs showed that they accumulate in the endocytic vesicles of APCs more slowly than NPs^+^, but that chloroquine pre-treatment did not reduce endocytosis by non-phagocytic cells, both of which suggest that endocytosis pathways differ from those of NPs^+^ [45]. Nevertheless, SMBVs are still able to efficiently deliver antigens within different cell lines, and no difference in the delivery of the GST-e4 fusion protein in APCs was observed when compared with NPs^+^. Interestingly, the in vitro CD4^+^ T-cell response to GST-e4 was greater when SMBVs were used than when NPs^+^ delivered the protein. In another study, SMBVs were also shown to improve the delivery of the fusion protein IE1-pp65 (resulting from the binding of the IE1 and matrix pp65 proteins from the hCMV) within human peripheral blood mononuclear cells (PBMCs) [46]. This greater antigen delivery increased both the proliferation and IFN-γ secretion from anti-IE1 CD8^+^ T-cells and enhanced the cytotoxic activity of anti-IE1 HLA-DR3-restricted CD41 T-cell clones [46].

Notably, SMBVs can also deliver oligonucleotides within cells to improve cancer therapies based on antisense nucleic acid which inhibit expression of oncogenes. An antisense ODN was designed to inhibit erbB-2 mRNA translation then tested in two cell models overexpressing the proto-oncogene protein P185 erbB-2 [41]. Again, SMBVs exhibited a 100% encapsulation efficiency when mixed with 10% (*w*/*w*) ODN, and the formulation induced a complete inhibition of cell growth during 120 h of treatment.

#### 4.1.3. SMBVs as a Delivery System for Vaccines

- Rabies vaccine

When SMBVs were loaded with rabies glycoprotein and ribonucleoprotein and administered to mice subcutaneously, they provoked a greater production of serum antibodies with a similar antibody profile compared with an administration of free antigens [41]. These results confirm that SMBVs enhanced the antigen immunogenicity but without any intrinsic immunomodulatory feature. The results also showed that SMBVs loaded with an experimental rabies vaccine, made with an inactivated virus, enhanced the protection against rabies infection compared to administration of the free rabies vaccine.

- Meningococcal (MenC) vaccine

A different study examined the use of SMBVs encapsulating a MenC vaccine and administered to mice nasally [41]. The formulation induced a secretion of anti-MenC IgG in the serum and anti-MenC secretory IgA (sIgA) in nasal washes. Moreover, the addition to the formulation of a nontoxic, heat-labile enterotoxin mutant (LTK63) of Escherichia coli as an adjuvant did not improve the anti-MenC immune response compared with the SMBV-MenC vaccine. This result confirmed the efficiency of SMBVs as a mucosal delivery system.

- Influenza clinical trials

Following these observations, SMBVs were used in a phase I human clinical trial against influenza infection. An influenza vaccine was prepared with three split viral strains encapsulated into SMBVs. A randomized, placebo-controlled, double-blind phase I study was performed with increasing vaccine doses (7.5, 15 and 30 µg) to evaluate the safety and immunogenicity of the formulation [41]. Except for nasal dripping in the 2 days following the first administration, no adverse effects or intolerance were observed with the different antigen doses, even at 8 mg of SMBVs. The immunogenicity was evaluated by measuring mucosal sIgA and serum hemagglutination inhibition (HAI) against each viral strain. Significant increases in antibody secretion were observed at all vaccine doses, with the greatest secretion observed in the 30 µg antigen group.

A second phase I human clinical trial assessed a different formulation. The vaccine was prepared with the hemagglutinin and neuraminidase from three influenza virus strains, associated with LTK63 as a mucosal adjuvant, and both were encapsulated in SMBVs. A randomized, placebo-controlled, double-blind phase I study was performed with increasing LTK63 doses (7.5, 15 and 30 µg) to evaluate the safety and immunogenicity of nasal administrations compared with intramuscular injections [47]. The encapsulation of antigens and 30 µg of LTK63 in SMBVs enhanced both the serum IgG responses and the nasal sIgA response against the virus strains.

Hence, SMBVs have been shown to be a versatile delivery system capable of being loaded with a range of antigens and molecules and delivering these treatments across mucosal barriers thanks to their structure and physicochemical characteristics. This technology, mimicking the behavior of a live virus, has demonstrated its potential to ensure the nasal delivery of vaccines, thanks to its ability to load and stabilize antigens, and to deliver them to the NALT, without inducing significant side effects.

### 4.2. NPL

Cationic maltodextrin NPs with a lipid core (NPLs) are similar to SMBVs, but anionic phospholipids are embedded within their structure (Figure 1). The aim in producing this variation of NPs^+^ was to mimic the physico-chemical characteristics of capsid viruses such as human rhinovirus [48]. Indeed, these 100 nm viruses can cross mucus layers and reach mucosal surfaces more efficiently that enveloped viruses, thanks to their external capsid protein surfaces densely coated with equal proportions of cationic and anionic charges, and with no hydrophobic zones exposed on their surface [49,50]. To produce NPLs, anionic dipalmitoyl-phosphoglycerol (DPPG) was incorporated within NPs^+^.

While incorporated within the maltodextrin core, DPPG can nevertheless interact with the environment. Studies performed on the interaction with complement proteins showed that the amount of complement proteins adsorbed was reduced with the gradual DPPG incorporation in the NP^+^ core. Moreover, with 70% (*w*/*w*) of incorporated lipids, NPLs behave exactly like uncharged, neutral maltodextrin NPs, while NPs^+^ bind strongly to the proteins. Similarly, a study carried out on respiratory tract mucus showed that the binding of NPLs to mucins decreased with increasing amounts of incorporated DPPG [51]. As a result, NPLs behave like zwitterionic NPs, despite their cationic surface charge, and are hence better vectors than NPs^+^ for crossing the mucosal barrier [52,53].

#### 4.2.1. Loading of Proteins and Molecules

Similar to SMBVs, the dual composition of NPLs permits a great loading capacity for molecules with different physicochemical features: as observed for NPs^+^, the cationic polysaccharidic scaffold can encapsulate anionic molecules and proteins, while the DPPG core can adsorb cationic and hydrophobic molecules.

NPLs can be loaded with up to 200% of their weight of proteins, despite the steric hindrance of the phospholipid core [54]. Measurements of the surface charge during loading indicated that proteins are fully encapsulated until 100% *w*/*w* loading is reached, and thereafter the antigens are associated with the surface of the NPL.

The encapsulation of more complex antigens was also evaluated using pathogenic total extracts (TE), which are crude extracts from killed pathogens containing a heterogeneous mixture of all the proteins, lipids and glucids of different sizes and solubilities. Studies performed on *Toxoplasma gondii* TE showed that 100% of the proteins were associated with the NPLs when mixed at a 1/1 weight ratio [55]. Other studies conducted with *E. coli* TE or with split viruses confirmed a 100% encapsulation with no release of the antigen over time [54,56].

Thanks to their lipid core, NPLs can also encapsulate hydrophobic molecules by means of post-loading association. Diminazene (DMZ), a trypanocidal drug which has only limited efficacy owing to its instability, was formulated with NPLs. While no association occurred with the NPs^+^, the addition of DPPG increased the drug encapsulation, with a maximum binding when NPLs contained 70% (*w*/*w*) of lipids. NPLs could load up to 4% (*w*/*w*) of the drug, with no aggregation nor drug release observed for at least 6 months in water. Finally, the encapsulation in NPLs protected the drug from oxidation, contrary to the free drug, and notably improved its trypanocidal efficiency when incubated with parasite cultures.

#### 4.2.2. Cell Interaction and Antigen Delivery

While NPLs are zwitterionic in behavior, they exhibit a cationic surface charge and are therefore strongly endocytosed. However, the DPPG core was shown to impact NPL behavior after cell uptake: they are indeed endocytosed more slowly by airway mucosal cells than NPs^+^, but deliver their cargo into the cytosol in a more efficient way. This suggests that the cytosolic delivery occurs after an endosomal escape induced by the DPPG, as shown for liposomal nanocarriers, which perturbs the organization of the endosomal membrane [57].

Moreover, NPLs were shown to be endocytosed more efficiently than other commonly developed delivery systems. A comparative study was performed with cationic and anionic liposomes (Lipo+ and Lipo−) and cationic and anionic PLGA NPs (PLGA+ and PLGA−) of the same size, ≤100 nm [58]. After 24 h of incubation, NPLs were shown to be endocytosed 30 times more than liposomes by airway epithelial cells, and 4 times more than PLGA+. They were also shown to be captured 46 times more than liposomes by dendritic cells and twice as much as PLGA+; meanwhile, in macrophages, NPLs were captured 65 times more than liposomes and 5 times more than PLGA+. These differences were even more pronounced when examining the minimum time of incubation required, confirming the efficiency with which NPLs are able to deliver antigens into airway cells. While NP^+^ uptake was only mediated by the clathrin pathway, the mechanisms underlying NPL uptake were linked to the triggering of the caveolae, clathrin and dynamin-mediated endocytosis pathways for all three cell lines cited above [58], confirming that the DPPG core influences NPLs’ interaction with their environment.

The NPLs’ interactions with the airway epithelial cells were studied in greater depth using a transwell model. This model can mimic a joint epithelium and allows the evaluation of transcytosis and paracellular transfer. It was observed that the incubation with NPLs did not disrupt the tight junctions, and that NPLs do not cross the epithelium by transcytosis nor by paracellular passage [55]. Moreover, all NPLs were found in the apical pole of the cells, even hours after the incubation, suggesting that NPLs, similarly to NPs^+^, end up being exocytosed from the cells. These results were confirmed in vivo, as no particles were found below the nasal epithelium or in the lymph nodes after a nasal administration.

**Figure 3:**
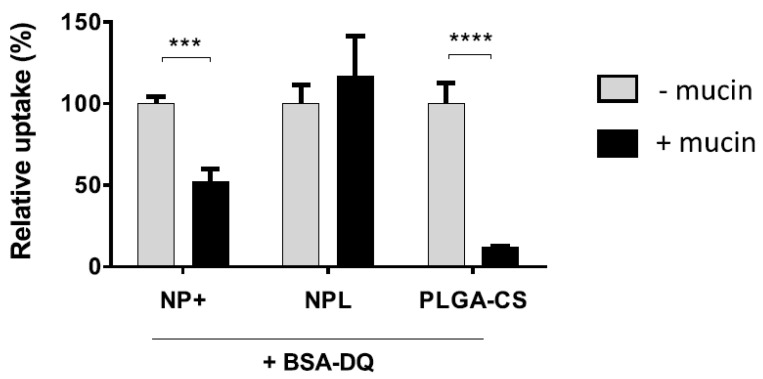
Intracellular delivery of BSA-DQ by each nanoparticle type in airway epithelial cells (H292) in both the absence and presence of mucins [51]. Dye-quenched BSA (BSA-DQ) was associated with each particle, and the NP formulations were mixed with mucins (mass ratio 1:1). The complexes were then incubated with airway epithelial cells (H292). Uptake measurements were carried out by flow cytometry. Statistical comparison made by Two-way ANOVA, *** *p* < 0.001, **** *p* < 0.0001. PLGA-CS: PLGA NPs covered with chitosan (CS). Reprinted with permission from [51]. Copyright 2020, American Chemical Society: Washington, DC, USA.

Finally, NPLs were found to cross the mucus layer surrounding epithelial cells, in contrast with NPs^+^, thanks to their anionic core (Figure 3). Mucus gel is made from hydrated mucins, which are proteins exhibiting anionic and hydrophobic zones, and which can strongly bind hydrophobic and cationic materials. The presence of mucins was consequently found to decrease the NP^+^ uptake by epithelial cells by 50%. However, no such influence was observed for NPL uptake [51]. As observed for the consumption of complement proteins, the addition of DPPG within NPLs improved their mobility in airway mucus by reducing their association with mucins. As with zwitterionic NPs, the presence of both cationic and anionic charges on the NP surface facilitates their diffusion in the mucus gel by reducing electrostatic interactions, while allowing interactions with the cell membrane and hence their endocytosis.

This mucopenetration and subsequent uptake are the key features that allows NPLs to exhibit a longer mucosal residence time compared to other NP delivery systems [58].

#### 4.2.3. Antigen Delivery

Several studies have described how NPLs can deliver antigens into APCs and airway epithelial cells in a very efficient manner. They were shown to deliver ovalbumin more efficiently than NPs^+^, probably thanks to the lipidic core promoting the endosomal escape [59]. But NPLs can also deliver complex antigens such as bacterial, parasitic and total virus extracts as a result of a strong protein encapsulation [54,56]. The comparative study carried out with liposomes and anionic PLGA NPs showed that NPLs increase the delivery of proteins within epithelial and immune cells [58].

Studies were conducted to establish whether these nanoparticles had an adjuvant effect on airway epithelial cells or if they acted purely as a delivery system. Interestingly, NPLs were proven to be intrinsically inert and to induce no inflammation or immunomodulation when incubated with airway epithelial and immune cells [54]. In contrast, lipid nanoparticles (LNPs), polymeric NPs or virus-like particles (VLPs) were shown to trigger innate immunity by themselves, via the activation of the inflammasome, leading to potential side effects [60,61,62,63]. Here, antigen delivery by NPLs was shown to trigger the innate immune response by epithelial cells and APCs, highlighted by the secretion of chemokines and Th1/Th17-orientated cytokines. NPLs therefore act purely as an antigen delivery system.

#### 4.2.4. NPLs as a Delivery System for Vaccines

- Safety

Despite being highly endocytosed, NPLs do not induce any damage to cell membranes, in contrast with numerous cationic NPs described in the literature. Geno-toxicological studies showed that they do not induce any DNA damage [64]. In rats, the nasal administration of a high NPL dose (8 mg/kg/day) for 2 consecutive days induced no side effects whatsoever, providing strong evidence that NPLs are suitable for nasal administration. They have since been used with success in several pre-clinical models as an antigen delivery system for nasal vaccines. This innocuity prevents eventual excessive local inflammation in the mucosal application site, which is often observed for inorganic NPs and other cationic NPs, as well as for NP-based vaccines administered with adjuvants [18,65,66]. Similarly to SMBVs, NPL nose-to-brain passage has never been observed in animal models [41,55].

- Toxoplasma gondii vaccine

**Figure 4:**
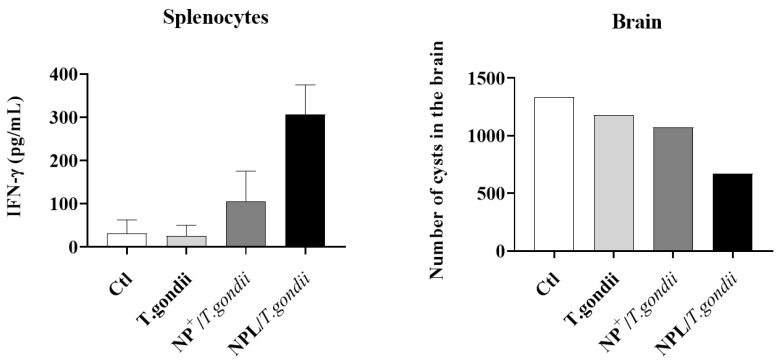
Cellular immune responses and protection against *T. gondii* infection [72]. Two mice were immunized 3 times with either free *T. gondii* or with *T. gondii* loaded into NPs^+^ or NPLs. The concentration of IFN-y (pg/mL) in supernatants of cultured splenocytes obtained from each group of treated mice (**left**). Mice were infected with *T. gondii* tachyzoites and the number of cysts in their brains was quantified (**right**).

In-depth studies have been carried out on a vaccine against *Toxoplasma gondii* infection, for which there is no effective vaccine [67,68]. This parasite infects all warm-blooded species and is responsible for numerous complications in humans, including cerebral diseases owing to the formation of cysts in the brain, blindness when the cysts reactivate in the eye, and spontaneous abortion upon infection of non-immunized, pregnant women [69,70]. In a congenital mouse model, a nasal administration of killed parasites associated with NPLs (NPL/*T. gondii*) was shown to protect 100% of animals after a challenge and protected fetuses from vertical transmission of the infection. This protection was mediated by the induction of a strong, systemic Th1/Th17 immunity against the parasite. Moreover, in a congenital ewe model, the nasal immunization of pregnant ewes protected the fetus from vertical transmission of the infection as well. The newborns were all viable and had fewer cerebral and ocular cysts compared with the other groups [55]. Further studies demonstrated that NPs^+^ were less efficient compared with NPLs in inducing a protective Th1 immune response against the parasite, confirming the importance of the DPPG core for the delivery system’s efficiency (Figure 4, [71]).

Based on these promising preclinical trials, a vaccination campaign to protect animals from lethal infection was initiated in several French zoological parks in late 2017. Some species, such as marsupials, lemurs and monkeys, are indeed very likely to develop a lethal infection, and many of these animals are endangered species [72]. When maintained in captivity, the parasite can be transmitted by the ingestion of contaminated food or water, but also via the dissemination of contaminated feline feces [73,74]. Saimiri subspecies were found to be particularly vulnerable, and can die of liver, brain, and lung failure only a couple of days after infection [75]. Several outbreaks have occurred in zoos during the last decade, leading to the loss of more than 75% of the infected animals [76,77,78]. French zoos were the first to participate in vaccination campaigns against this parasite, with the animals receiving a nasal prime/boost vaccination followed by three heterologous nasal/subcutaneous boosts in an attempt to broaden the immune protection. The animals developed an early Th1 immune response, followed by a humoral response engendered by the addition of subcutaneous administration. Thanks to this immune response, while deaths related to *T. gondii* infection occurred in unvaccinated animals, all of the vaccinated animals have survived. Recently, a less invasive homologous vaccination protocol was established, consisting of two nasal administrations at one-month intervals, followed by a nasal boost at 6 months [79]. A specific, memory T-cell immunity was observed after two administrations in all the squirrel monkeys thus treated, and this immunity lasted for at least 6 months, suggesting that this immunization schedule is sufficient to induce a protective immune response. This vaccination campaign is ongoing and has been widened to include other zoological parks in Europe and South America.

- Canine Leishmaniasis immunotreatment

Regarding the ability of NPLs to induce a Th1/Th17 immune response after nasal administration, studies were conducted to develop an immunotreatment against canine Leishmaniasis (CanL). CanL is caused by Leishmania subspecies, and poses a major threat to the canine population in South America. Current drugs used to treat CanL often fail to clear the infection, while inducing numerous side effects [80,81]. As CanL is an immunomodulated disease, the use of immunotreatments should strengthen the deficient immune response of infected dogs [82,83]. The vaccine was composed of killed *Leishmania infantum* parasites associated with NPLs (NPL/L.inf). A 3-month study was conducted on naturally infected dogs in Brazil. Two nasal administrations, two weeks apart, induced a decrease in the parasite burden in both the skin and the bone marrow, and also sharply decreased the infection-related humoral response [84]. A slight improvement in the clinical scoring was also observed, although the dogs had other infections, and no side effects were reported. Overall, the immunotreatment was safer and at least as efficient as the current Brazilian chemotherapy protocol, based on 28 oral administrations of milteforan over a period of one month [84,85].

- Influenza vaccine

Studies were conducted to evaluate whether a nasal vaccination could induce both protection and prevent the viral transmission of influenza. The nasal administration of a split-Udon virus loaded into NPLs increased the serum IgG titers and provoked a concomitant decrease in the lung viral titers after a non-lethal challenge. Moreover, the vaccine was also shown to prevent the transmission of the virus. NPLs were also loaded with a fusion protein containing three copies of the viral matrix protein 2 ectodomain (M2e) [86]. The nasal administration protected all of the mice in receipt of this formulation from a lethal challenge, once again by triggering a Th1/Th17 systemic immune response associated with an IgG serum response and an increased sIgA secretion in the lungs.

These results highlight the interest in developing a nasal vaccine against SARS-CoV-2 infections using these particles as a delivery system. This could trigger the secretion of sIgA in the airway along with a systemic Th1/Th17 immune response, inducing protection against both the infection and the transmission [87,88].

Overall, it can be seen that the nasal administration of parasitic antigens triggers a Th1/Th17 immune response, whereas viral antigens loaded in NPLs lead to the establishment of both cellular and humoral (local and in serum) immune responses. It has been established that NPLs act as a pure antigen delivery system, with no intrinsic immunomodulation., and the immune response triggered therefore depends solely on the epitopes exposed by the antigens. This compelling body of work suggests that NPLs are a highly versatile delivery system with enormous potential in the development of vaccines against a wide range of pathogens.

## 5. Conclusions

Nasal vaccination is a very attractive route for several reasons, as it avoids the need for an injection and can induce protection against both the infection and transmission of pathogens. Studies to date that have used attenuated pathogens have had only limited success owing to the need to infect the mucosa, leading to potential side effects. The use of inactivated vaccines is limited to delivery systems capable of crossing the mucosal membrane and delivering the antigens into the immune cells without toxicity, and nanoscale formulations exhibit all of these capabilities. Maltodextrin nanoparticles can further improve the delivery of inactivated vaccines through the airway mucosa compared with other NPs, and have been conclusively demonstrated to safely induce a long-lasting, protective response against influenza virus and *Toxoplasma gondii* infection. Moreover, the addition of lipids to NPs^+^ creates vectors (NPLs) with improved properties for vaccine delivery. These nanoparticles, which can be used without an adjuvant, are a highly promising tool for the development of mucosal vaccines with a very wide range of applications.

## Figures and Tables

**Table 1:** Summary of the structural and biological differences between NPs+, SMBVs and NPLs.

	NPs+	SMBVs	NPLs
Structure	Cationic maltodextrin scaffold	Cationic maltodextrin scaffold covered by cationic phospholipids	Cationic maltodextrin scaffold loaded with anionic phospholipid
Size	60–100 nm	50–90 nm	60–100 nm
Surface charge	Cationic	Neutral	Cationic
Ability to load protein	Yes	Yes	Yes
Ability to load hydrophobic drugs	No	Yes	Yes
Uptake ^1^	Fast	Slow	Fast
Main endocytosis pathway ^1^	Clathrin	Caveolae	Clathrin/Caveolae
Exocytosis ^1^	Yes	N.A.	Yes
Cytoplasmic protein delivery ^1^	No	Yes	Yes
Mucus interaction	Mucoadherence	Mucopenetration	Mucopenetration
Immune response after mucosal administration	Th2 > Th1	Th1 ≈ Th2	Th1/Th17 > Th2

^1^ On airway epithelial cells.
